# Socio-economic characteristics and career intentions of the WiSDOM health professional cohort in South Africa

**DOI:** 10.1371/journal.pone.0223739

**Published:** 2019-10-21

**Authors:** Laetitia Charmaine Rispel, Prudence Ditlopo, Janine Anthea White, Duane Blaauw

**Affiliations:** 1 Centre for Health Policy & Department of Science and Innovation (DSI) and National Research Foundation (NRF) Research Chair, School of Public Health, Faculty of Health Sciences, University of the Witwatersrand, Johannesburg, Parktown, South Africa; 2 Centre for Health Policy, School of Public Health, Faculty of Health Sciences, University of the Witwatersrand, Johannesburg, Parktown, South Africa; 3 School of Public Health, Faculty of Health Sciences, University of the Witwatersrand, Johannesburg, Parktown, South Africa; Aga Khan University, KENYA

## Abstract

**Background:**

The human resources for health (HRH) crisis and dearth of research on the health labour market in South Africa informed the WiSDOM (Wits longitudinal Study to Determine the Operation of the labour Market among its health professional graduates) cohort study. The study aims to generate new knowledge on the career choices and job location decisions of health professionals in South Africa.

**Methods:**

WiSDOM is a prospective longitudinal cohort study. During 2017, the first cohort for each of eight professional groups was established: clinical associates, dentists, doctors, nurses, occupational therapists, oral hygienists, pharmacists and physiotherapists. These cohorts will be followed up for 15 years. For the baseline data collection, each final year health professional student completed an electronic self-administered questionnaire (SAQ), after providing informed consent. The SAQ included information on: demographic characteristics; financing of training; reasons for choosing their profession; and their career intentions. We used STATA^®^ 14 to analyse the data.

**Results:**

We obtained an 89.5% response rate and 511 final year health professional students completed the baseline survey. The mean age of all participants was 24.1 years; 13.1% were born in a rural area; 11.9% and 8.0% completed their primary and secondary schooling in a rural area respectively. The health professional students came from relatively privileged backgrounds: 45.0% had attended a private school, the majority of their fathers (77.1%) had completed tertiary education, and 69.1% of their mothers had completed tertiary education. Students with higher socio-economic status (SES Quintiles 3–5) made up a larger proportion of the occupational therapists (77.8%), physiotherapists (71.7%), doctors (66.7%), and dentists (64.7%). In contrast, individuals from SES Quintiles 1 and 2 were over-represented among the clinical associates (75.0%), oral hygienists (71.4%), nurses (61.9%), and pharmacists (56.9%). Almost one quarter (24.9%) of cohort members indicated that they had partly financed their studies through loans. Although 86.3% of all cohort members indicated that they plan to stay in their chosen profession, this ranged from 43.2% for clinical associates to 100% for dentists.

**Conclusions:**

WiSDOM has generated new knowledge on health professional graduates of a leading South African University. The results have implications for university selection criteria and national health workforce planning.

## Introduction

Investment in the health workforce or human resources for health (HRH) is essential for strong and resilient health systems [1] and is a prerequisite for universal health coverage (UHC) [2]. In 2016, the United Nations High-Level Commission on Health Employment and Economic Growth proposed research on, and analysis of, health labour markets as part of wide-ranging recommendations to ensure that all countries have sufficient numbers of competent, equitably-distributed HRH for UHC [3]. A health labour market is defined as “a dynamic system comprising two distinct but closely related economic forces: the supply of health workers and the demand for such workers, whose actions are shaped by a country’s institutions and regulations” [4]. However, there is a dearth of research on health labour markets in low- and middle-income countries (LMICs) [4]. Such labour market research can generate new knowledge on the factors behind health workforce constraints, inform health workforce training decisions and contribute to the design of more effective policies and/or HRH interventions [5].

South Africa has at least 4 health professionals (doctors, nurses and midwives) per 1000 population, which is well above the minimum World Health Organization (WHO) norm of 2.5 per 1000 [6]. It also has well-established training institutions, skilled health workers, and relatively effective professional regulation [7]. Notwithstanding these strengths, there is a health workforce crisis in the country. This crisis manifests as geographical inequity of HRH, mal-distribution between the public and private health sectors, ineffective leadership, management and governance, staff shortages [8, 9], unprofessional behaviour and poor staff motivation [6]. These challenges compromise the quality of patient care and contribute to the sub-optimal performance of the health system [10]. At present, the government’s main reform aimed at improving the poor performance of the health care system is the establishment of a National Health Insurance (NHI) system [11]. The NHI proposals are intended to improve progress towards UHC through major changes in national health financing, the organisation of the health system and the role of the private health sector [12]. However, the implementation of the NHI will be difficult without addressing HRH constraints and without evidence on how health workers respond to policy changes [13].

Notwithstanding the substantial and growing body of literature on the health workforce, the vast majority of HRH studies consist of case-studies [14, 15] or use cross-sectional study designs [16–20]. Although these cross-sectional studies provide useful insights, they have a number of important limitations: they only include health professionals that have remained in the health system; they do not provide information on dynamic changes over time; and they cannot relate cause (such as a new HRH policy) and their effects (e.g. increased health worker attrition or retention) [21]. Furthermore, national HRH information systems are weak or non-existent, especially in LMICs [22–24], while health workforce data from regulatory authorities vary considerably and are often limited to elementary socio-demographic information [5, 25].

Longitudinal study designs on the health workforce remain a novelty in HRH research and provide several advantages over cross-sectional studies [21, 26, 27]. These studies have the potential to examine the unique aspects of health labour markets, which include government policy interventions to influence demand and/or supply of health workers, and individual health worker preferences about work, leisure, family and lifestyle, and incentives embedded in a country’s health system financing and organisation [28]. However, the majority of these studies are conducted in high-income countries [25, 29–33]. Nonetheless, there has been an encouraging increase in the use of HRH longitudinal study designs in LMICs, including the Chinese Nurses’ Early eXit sTudy (NEXT) to investigate intention to leave and working conditions [34], the Thai Nurse Cohort Study to generate evidence on the workforce dynamics and health status of Thai nurses [35], and the Nurses Cohort Study in South Africa to determine the career choices and job location decisions of 377 nurses in the cohort [36]. However, the majority of these longitudinal cohort studies tend to focus on one category of health professionals, and hence significant knowledge gaps remain.

There is insufficient data on the health labour market, and a dearth of knowledge on the long-term career choices and job location decisions of health professional graduates in South Africa. We aimed to address this gap by establishing a cohort of eight professional groups that graduated at the University of the Witwatersrand (Wits) in 2017. Wits University is located in Johannesburg, South Africa, the vibrant, culturally diverse and leading commercial city on the African continent.

Given the importance of the health workforce to achieving UHC, and to improving the performance of the South African health system, this paper analyses the baseline characteristics of the WiSDOM (**Wi**ts longitudinal **S**tudy to **D**etermine the **O**peration of the labour **M**arket among its health professional graduates) cohort study. The WiSDOM study aims to examine the career choices and job location decisions of Wits health professional graduates over a period of 15 years. The WiSDOM study is critical in light of the current health sector reforms in South Africa [12] and the global emphasis on research and the importance of investing in the health workforce [3].

## Methods

### Study setting

Wits University was established in 1922, and is an internationally ranked, leading, research intensive university with a global footprint [37, 38]. The University has a student body of 36 500 and comprises five faculties, of which the Faculty of Health Sciences (FHS) is one. Although medical and basic science was first taught in 1912 before the formal establishment of the University in 1922, the FHS celebrates its centenary in 2019 because the new Hospital Hill Medical school was designed in 1919 [39]. The FHS plays an important, strategic role in health service delivery and in the education of health professionals in South Africa and in the Africa region. Each year, the FHS graduates around 600 health professionals, who are sought after nationally, regionally and globally [38]. Similar to other universities in South Africa, the FHS has specific selection policies in an effort to ensure that the health professional student intake is more representative of the broader population [40]. The FHS is a leading research hub on the African continent and in the world, with the research impacting directly on improving the lives of communities in South Africa [38].

### Study design

**Fig 1:**
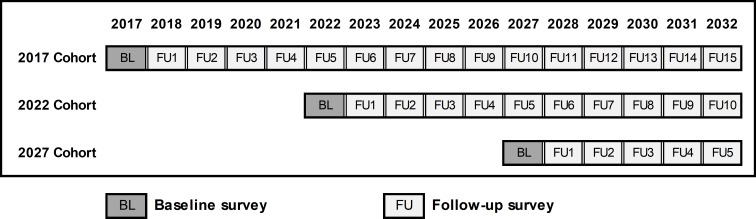
Overview of WISDOM study design.

WiSDOM is a prospective longitudinal cohort study. During 2017, the first cohort for each of eight professional groups was established: clinical associates, dentists; doctors; nurses, occupational therapists, oral hygienists, pharmacists and physiotherapists. These cohorts will be followed up for 15 years. More details of the WiSDOM study are available on the study website [41]. An overview of the WiSDOM’s study design is shown in Fig 1. A single cohort would fail to capture changes in Wits graduates over time but following up annual cohorts would be unmanageable. Hence, we have decided to add a new cohort of graduates every five years with the establishment of the next cohorts planned for 2022 and 2027.

### Study population

The study population consists of the eight health professional categories trained in the Wits Faculty of Health Sciences: clinical associates, dentists, doctors, nurses, occupational therapists, oral hygienists, pharmacists and physiotherapists.

Clinical associates (also called physician assistants or clinical officers in other countries), are mid-level healthcare workers, who undergo a three-year, full-time Bachelor of Clinical Medical Practice degree that aims to develop sound knowledge of the medical and clinical sciences [42]. At the end of the training, they are registered with the Health Professions Council of South Africa (HPCSA) as clinical associates. Clinical associates are not required by law to do internship or compulsory community service after graduation.

The Bachelor of Dental Science is a five-year, full-time degree leading to registration as a dentist with the HPCSA. Dentists are expected to complete one year of compulsory community service after graduating, and prior to registration with the HCPSA [42].

The Bachelor of Medicine and Bachelor of Surgery (MBBCh) under-graduate medical degree is a six-year, full-time degree for school leavers, leading to registration as a medical doctor. Graduates are expected to complete two years of internship and one year of compulsory community service, prior to registration as a medical doctor with the HPCSA [42].

The Bachelor of Nursing is a four-year degree which prepares nursing professionals for nursing and midwifery practice [42]. Graduates are required to do one year of compulsory community service, prior to registration with the South African Nursing Council.

The Bachelor of Science in Occupational Therapy is a four-year professional degree which focuses on the Science of Occupation and Occupational Therapy [42]. Graduates are required to do one year of compulsory community service prior to registration as an occupational therapist with the HPCSA.

The Bachelor of Oral Health Sciences, which focuses on the prevention of oral disease and the maintenance of good oral hygiene, is a three-year, full-time degree that leads to registration as an oral hygienist with the HPCSA [42]. Graduates are not required to do internship or compulsory community service.

The Bachelor of Pharmacy is a four-year, full-time degree [42]. Graduates are expected to do one year of internship, and one year of compulsory community service, prior to registration as a pharmacist with the South African Pharmacy Council.

The Bachelor of Science in Physiotherapy is a four-year, full-time degree. Graduates are required to do one year of compulsory community service, prior to registration as a physiotherapist with the HPCSA [42].

### Sampling approach

All 2017 graduating students were invited to participate in the study, and no sampling was done.

### Communication and consultation

We embarked on an extensive consultation process to: elicit stakeholder inputs on the study; determine best communication strategies with potential cohort members; get ideas on appropriate incentives for voluntary participation; obtain stakeholder support for the study; obtain schedules of the final year academic programme; and to obtain permission and support for the data collection as part of the academic programme. Our primary stakeholders were final year students whom we reached via their student representative councils, and the final year class representatives. Our secondary stakeholders were academic course coordinators, lecturers, heads of clinical and academic departments, and course administrators. We also consulted with the FHS executives or office bearers, and we approached government officials, alumni and motivational speakers for talks following the data collection process.

Following 200 person-hours of consultation, we developed a three-minute video, in which the student representatives of cohort members featured. We used the video, posters and customised adverts to encourage voluntary participation of all potential cohort members, and asked class representatives to distribute the notices via social media as well.

### Development of data collection tool

We adapted the baseline self-administered questionnaire (SAQ) used in the South African Nurses Cohort Study [36] and customised the tool for each of the health professional cohorts. The questionnaire consisted of eight sections and covered the following areas: detailed contact information (own, parents, spouse, friends); demographic characteristics (age, gender, marital status, children, place of birth); financing of training; views on FHS selection criteria and transformation initiatives, and the Wits University experience; attitudes to community and rural service, altruism and public service motivation; career intentions (employment, profession, specialisation); and job location intentions (country, sector, geographical area, facility type). This paper focuses on socio-demographics, financing, socio-economic status, career motivations and intentions. Other aspects from the SAQ will be covered in future papers. An analysis of the socio-economic status (SES) of students is important because of debates about subsidised higher education in South Africa, university initiatives to accept more disadvantaged students, and because SES may influence future career and job choices.

### Pilot study

Three of the research team members completed the survey for all eight professional groups on REDCap (Research Electronic Data Capture) [43], a secure, web-based application designed to support data capture for research studies, to check for errors. Seven students in the penultimate year of study -from the nursing, medicine and clinical associate classes- completed the questionnaire to test the clarity of questions and the time taken for completion. These students made valuable inputs that led to the addition of three questions. The questionnaire took an average of 15 minutes to complete.

### Data collection

We conducted the baseline survey between July and September 2017, with separate data collection sessions for each of the eight professional groups. In consultation with course coordinators and student representatives, we identified an appropriate timeslot in the final year academic programmes for each group, on days when the students were expected for lectures at the Wits Health Sciences Campus.

We informed students about the study and data collection sessions through class announcements, the student councils, pamphlets and public notices. At the baseline survey session, all participating students were given the study information sheet. After obtaining informed consent, each final year health professional student completed an electronic self-administered questionnaire (SAQ) on REDCap. Each data collection session was followed by a Department of Health talk on community service or internship and a motivational talk by young practitioners, who had completed their internship and/or community service. We arranged these talks at the recommendation of the various class representatives. These talks provided study participants with the opportunity to ask questions and served as a non-monetary incentive to participate in the baseline study. We also provided refreshments and gave each student a memory stick with the study logo to thank them for their participation.

Students who were unable to participate in the initial classroom data collection were given the opportunity to complete the SAQ online, in order to maximise the response rate.

### Data analysis

Following the closure of the electronic survey, we imported the data from REDCap into STATA^®^ 14 for analysis. Frequency tabulations were done to describe the socio-demographic characteristics of the study participants.

In addition to the individual socio-economic variables, we also constructed a composite socio-economic index to simplify comparisons of the relative socio-economic status (SES) of the Wits health professional graduates. The ten variables in the final index were: being born in an urban area; attended urban primary school; urban secondary schooling; mother has tertiary qualification; father has tertiary qualification; mother employed; father employed; attended private school; whether parents’ paid for university education; and received funding from the government financing scheme for disadvantaged students. The Cronbach’s alpha for these variables combined was 0.729 but we used principal component analysis (PCA) to derive an appropriately weighted final index. We used the scores of the first principal component, which explained 32.1% of the variance, as the SES index and to divide the cohort members (graduates) into socio-economic quintiles.

### Ethical considerations

The Human Research Ethics Committee (Medical) of the University of the Witwatersrand in Johannesburg provided ethical approval for the study (M170 550). The Dean of the Faculty of Health Sciences and the Deputy Registrar of Wits University also provided study approval. All participants received a detailed study information sheet, and provided written consent, via REDCap (Research Electronic Data Capture) a secure, web-based application designed to support data capture for research studies [43]. The front screen of the SAQ contained the approval button for written consent. Participants were unable to proceed with the survey, until they had indicated approval by pressing the ‘yes’ button. We adhered to standard ethical procedures of voluntary participation, respect, and confidentiality.

We informed all potential participants about the longitudinal nature of the study and that they will be contacted each year for the follow-up survey. We also informed them that their participation in this study is strictly voluntary and that they will be free to withdraw at any time.

## Results

We obtained an 89.5% response rate, and 511 final year health professional students completed the baseline survey.

### Demographic and social characteristics

The demographic and background characteristics of the WISDOM cohort are shown in Table 1. The majority of cohort members (55.2%) were medical students. The mean age of all participants was 24.1 years, with a range of 19.8 to 37.2 years. Women comprised the majority of WiSDOM participants (71.8%).

**Table 1:** Demographic and social characteristics of WISDOM cohort members.

Characteristic	CA		DT		MD		NS		OT		OH		PH		PT		Total	
	n	%	n	%	n	%	n	%	n	%	n	%	n	%	n	%	n	%
**Age** (median/mean)	21.3	21.9	24.4	25.4	24.5	25.2	22.6	23.0	22.4	22.6	21.1	21.3	22.2	22.7	22.6	22.9	24.0	24.1
<25	41	93.2	9	52.9	175	62.3	20	95.2	35	100.0	7	100.0	52	91.2	42	91.3	381	75.0
25–29	3	6.8	7	41.2	94	33.5	1	4.8		0.0	0	0.0	4	7.0	4	8.7	113	22.2
30–39	0	0	1	5.9	12	4.3	0	0.0	0	0.0	0	0.0	1	1.8	0	0.0	14	2.8
**Gender**																		
Female	38	86.4	13	76.5	181	64.2	17	81.0	36	100.0	7	100.0	36	62.1	39	84.8	367	71.8
Male	6	13.6	4	23.5	101	35.8	4	19.0	0	0.0	0	0.0	22	37.9	7	15.2	144	28.2
**Self-identified Race**																		
Black African	40	93.0	2	11.8	115	42.1	17	81.0	2	6.1	5	71.4	39	68.4	8	18.2	228	46.1
Coloured	0	0.0	1	5.9	7	2.6	0	0.0	0	0.0	0	0.0	1	1.8	6	13.6	15	3.0
Indian	1	2.3	10	58.8	45	16.5	0	0.0	7	21.2	1	14.3	15	26.3	7	15.9	86	17.4
White	2	4.7	3	17.z	104	38.1	4	19.0	24	72.7	1	14.3	2	3.5	23	52.3	163	32.9
Asian	0	0.0	1	5.9	2	1.7	0	0.0	0	0.0	0	0.0	0	0.0	0	0.0	3	0.6
**Married**																		
Yes	0	0.0	3	17.6	24	8.5	0	0.0	0	0.0	0	0.0	1	1.7	3	6.5	31	6.1
No	44	100.0	14	82.4	258	91.5	21	100.0	36	100.0	7	100.0	57	98.3	43	93.5	480	93.9
**Children**																		
Yes	5	11.4	1	5.9	9	3.2	1	4.8	0	0.0	0	0.0	2	3.5	0	0.0	18	3.5
No	39	88.6	16	94.1	273	96.8	20	95.2	36	100.0	7	100.0	56	96.5	46	100.0	493	96.5
**Born in South Africa**																		
Yes	42	95.5	17	100.0	250	88.7	19	90.5	35	97.2	7	100.0	54	93.1	43	93.5	467	91.4
No	2	4.5	0	0.0	32	11.3	2	9.5	1	2.8	0	0.0	4	6.9	3	6.5	44	8.6
**Area of birth**																		
Urban	38	86.4	17	100.0	247	87.6	17	81.0	35	97.2	6	85.7	41	70.7	43	93.5	444	86.9
Rural	6	13.6	0	0.0	35	12.4	4	19.0	1	2.8	1	14.3	17	29.3	3	6.5	67	13.1
**Area of primary schooling**																		
Urban	35	79.6	17	100.0	249	88.3	20	95.2	35	97.2	6	85.7	44	75.9	44	95.6	450	88.1
Rural	9	20.5	0	0.0	33	11.7	1	4.8	1	2. 8	1	14.3	14	24.1	2	4.4	61	11.9
**Area of secondary schooling**																		
Urban	38	86.4	17	100.0	260	92.2	21	100.0	35	97.2	6	85.7	49	84.5	44	95.6	470	92.0
Rural	6	13.6	0	0.0	22	7.8	0	0.0	1	2.8	1	14.3	9	15.5	2	4.4	41	8.0
**Type of schooling**																		
Public/ Government	34	77.3	10	58.8	140	49.6	16	76.2	13	36.1	5	71.4	41	70.7	22	47.8	281	55.0
Private	10	22.7	7	41.2	142	50.4	5	23.8	23	63.9	2	28.6	17	29.3	24	52.2	230	45.0
**Mother’s tertiary education**																		
Yes	21	53.8	11	64.7	198	74.7	12	57.1	23	63.9	3	50.0	40	71.4	28	60.9	336	69.1
No	18	46.2	6	35.3	67	25.3	9	42.9	13	36.1	3	50.0	16	28.6	18	39.1	150	30.9
**Father’s tertiary education**																		
Yes	17	77.3	11	73.3	195	81.6	11	68.8	27	79.4	1	33.3	27	69.2	27	64.3	316	77.1
No	5	22.7	4	26.7	44	18.4	5	31.2	7	20.6	2	66.7	12	30.8	15	35.7	94	22.9
**Completed other degree**																		
Yes	3	6.8	6	35.3	60	21.3	0	0.0	2	5.6	0	0.0	3	5.2	5	10.9	79	15.5
No	41	93.2	11	64.7	222	78.7	21	100.0	34	94.4	7	100.0	55	94.8	41	89.1	432	84.5

CA = clinical associate; DT = dentist; MD = medical doctor; NS = nurse; OH = oral hygienist; OT = occupational therapist; PH = pharmacist; PT = physiotherapist

In terms of geographical distribution, 13.1% of WiSDOM members were born in a rural area; while 11.9% and 8.0% completed their primary and secondary schooling in a rural area respectively. At the time of the survey, 15.5% of cohort members had a previous degree. Almost every second student (45.0%) had attended a private school, ranging from 22.7% for clinical associates to 63.9% for occupational therapists. The majority of their fathers (77.1%) had completed tertiary education and 69.1% of their mothers had completed tertiary education.

### Socio-economic status index

Table 2 shows the relative socio-economic status of the study participants. There were important differences across the professional groups. Students with higher socio-economic status (SES Quintiles 3–5) made up a larger proportion of the occupational therapists (77.8%), physiotherapists (71.7%), doctors (66.7%), and dentists (64.7%). In contrast, individuals from Quintiles 1 and 2 were over-represented among the clinical associates (75.0%), oral hygienists (71.4%), nurses (61.9%), and pharmacists (56.9%). The mean SES scores show a similar pattern with clinical associates, oral hygienists, pharmacists and nurses below the average.

**Table 2:** Composite socio-economic status of WISDOM cohort members.

SES Index	CA	DT	MD	NS	OT	OH	PH	PT	Total
Mean score	-1.03	0.63	0.20	-0.48	0.75	-1.03	-0.79	0.32	0.00
SES Quintiles (%)									
1 Lowest SES	43.2	5.9	15.2	38.1	5.6	28.6	39.7	10.9	20.2
2	31.8	29.4	18.1	23.8	16.7	42.9	17.2	17.4	20.0
3	9.1	11.8	23.0	19.0	19.4	28.6	15.5	34.8	21.3
4	6.8	29.4	25.5	19.0	33.3	0.0	22.4	13.0	22.5
5 Highest SES	9.1	23.5	18.1	0.0	25.0	0.0	5.2	23.9	16.0
Quintiles 1–2 (%)	75.0	35.3	33.3	61.9	22.2	71.4	56.9	28.3	40.1
Quintiles 3–5 (%)	25.0	64.7	66.7	38.1	77.8	28.6	43.1	71.7	59.9

CA = clinical associate; DT = dentist; MD = medical doctor; NS = nurse; OH = oral hygienist; OT = occupational therapist; PH = pharmacist; PT = physiotherapist

### Financing of training

**Fig 2:**
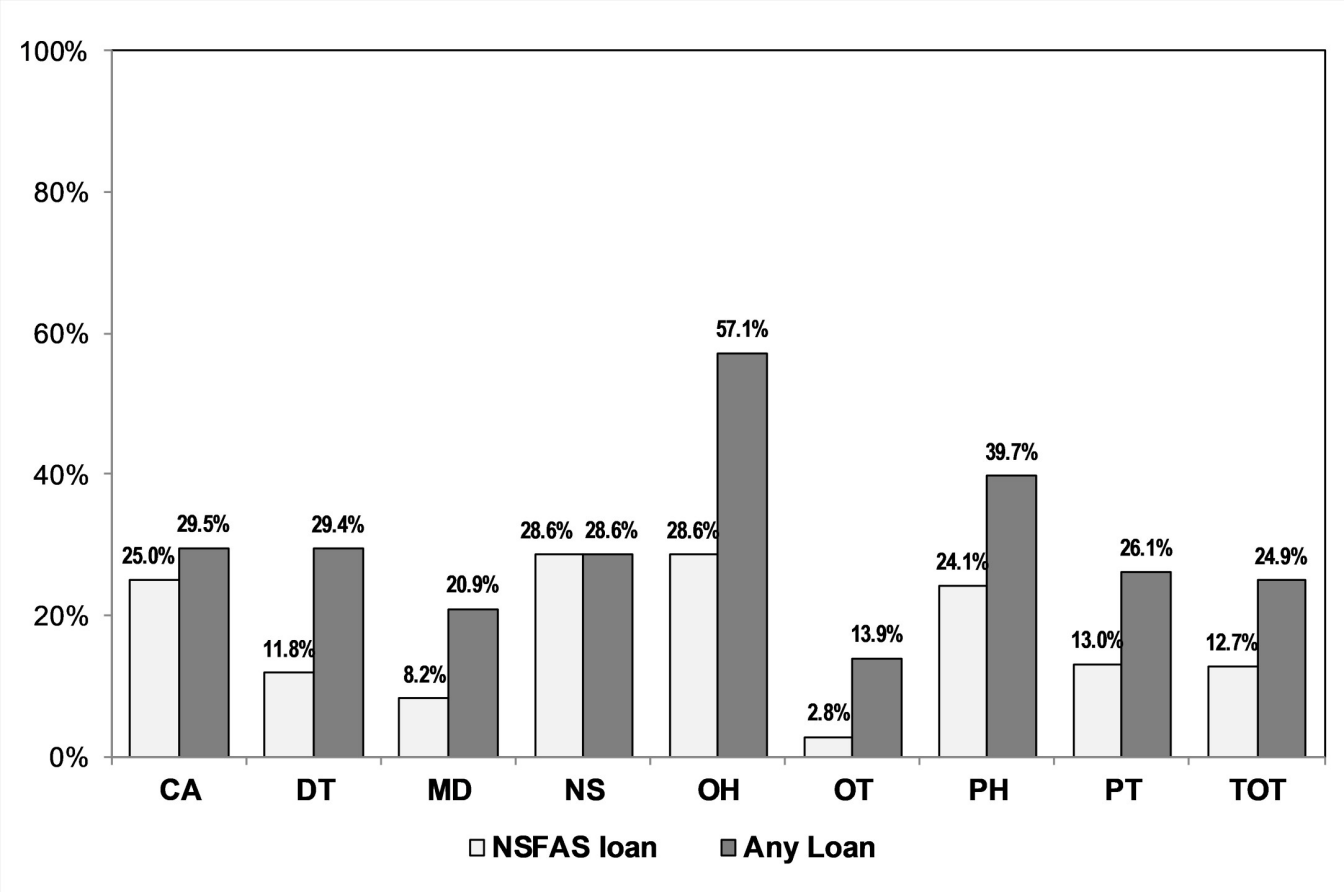
Loan financing of studies of WISDOM cohort members. CA = clinical associate; DT = dentist; MD = medical doctor; NS = nurse; OH = oral hygienist; OT = occupational therapist; PH = pharmacist; PT = physiotherapist; NSFAS = National Student Financing Aid Scheme.

Although our SES index shows the relatively privileged backgrounds of some cohort members, almost one quarter (24.9%) of cohort members indicated that they had financed their studies partly through loans (Fig 2), with 12.7% of all cohort members obtaining assistance through the government’s National Student Financial Aid Scheme (NSFAS). This is a government scheme provided to students at universities whose parents earn a combined income of R350 000 ($24 000; 1 USD = R15) per annum [44].

The groups with the highest proportion of loan financing were oral hygienists (57.1%), pharmacists (39.7%), clinical associates (29.5%) and dentists (29.4%). In concert with their SES, only 13.9% of occupational therapists had used some form of loan to finance their studies. NSFAS funding played a relatively small role in loan financing of occupational therapists (2.8%), and doctors (8.2%).

### Reasons for chosen profession

Table 3 shows the reported reasons for their chosen professions: 95.9% of all cohort members agreed with a statement that they want to help others, and this was consistent across the groups. Similarly, 87.1% agreed with a statement that they want to make a difference, ranging from 76.2% for nurses to 94.4% for occupational therapists. Although 81.0% agreed with a statement that their chosen career gives them job security, the highest proportions were for pharmacists (91.4%), physiotherapists (84.8%), dentists (88.2%) and doctors (86.2%). Only 47.7% of clinical associates agreed with this statement. Similarly, although 61.6% agreed with a statement that their chosen career pays good money, the highest proportions were for dentists (94.1%), pharmacists (81.0%) and doctors (75.9%). In contrast, only 4.8% of nurses and 9.1% of clinical associates agreed with the statement that their chosen profession pays good money.

**Table 3:** Reasons for chosen profession.

Reason	CA	DT	MD	NS	OT	OH	PH	PT	Total
My peers influenced me	2.3	11.8	14.5	14.3	13.9	0.0	12.1	17.4	13.1
My family influenced me	31.8	47.1	50.0	42.9	58.3	71.4	44.8	50.0	48.3
A role model inspired me	13.6	41.2	52.8	14.3	52.8	14.3	34.5	32.6	48.3
I want to make a difference in my community	81.8	82.4	89.4	76.2	94.4	85.7	84.5	82.6	87.1
I want to help others	93.2	94.1	97.2	95.2	97.2	100.0	91.4	95.7	95.9
It offers job security	47.7	88.2	86.2	66.7	66.7	71.4	91.4	84.8	81.0
It pays good money	9.1	94.1	75.9	4.8	16.7	14.3	81.0	56.5	61.6
There is a need for people with my degree in my community	86.4	82.4	79.1	57.1	91.7	57.1	84.5	84.8	80.6
It gives a stipend during training	31.8	29.4	9.9	23.8	5.6	0.0	17.2	21.7	14.5

CA = clinical associate; DT = dentist; MD = medical doctor; NS = nurse; OH = oral hygienist; OT = occupational therapist; PH = pharmacist; PT = physiotherapist

### Career intentions

**Fig 3:**
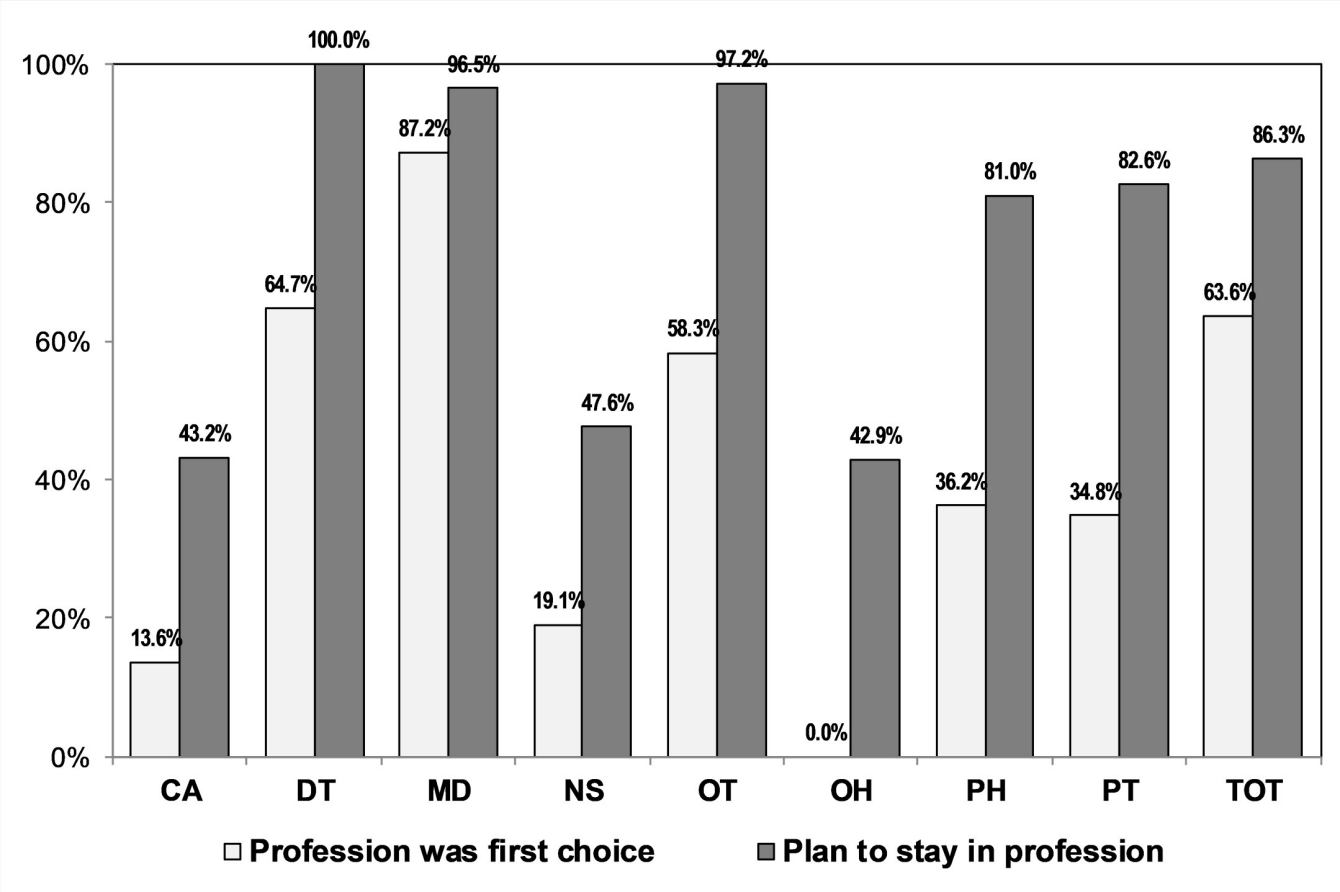
Choice of profession and intention to stay in profession. CA = clinical associate; DT = dentist; MD = medical doctor; NS = nurse; OH = oral hygienist; OT = occupational therapist; PH = pharmacist; PT = physiotherapist.

Fig 3 shows whether the career in which they graduated was a first choice of WiSDOM cohort members, and whether they intended to stay in that profession. 63.6% of cohort members indicated that the profession in which they graduated was their first choice-this ranged from 0.0% for oral hygiene students to 87.2% for medical students. Although 86.3% of all cohort members indicated that they plan to stay in their chosen profession, this ranged from 43.2% for clinical associates to 100.0% for dentists.

## Discussion

WiSDOM is a novel, pioneering cohort study in South Africa, and indeed, in Africa, that will observe the career intentions and job location decisions of multiple health professionals and the factors that influence their decisions over a period of 15 years. This paper has presented a comparison of the demographics, socio-economics, career motivations and intentions of the different health professional groups that constitute WiSDOM.

We obtained a high response rate (89.5%), indicating a representative sample of health professional graduates at Wits University, a major methodological strength of the study. Other studies demonstrated variable response rates. The Chinese NEXT study obtained a similar response rate of 89.95% [34], while the response rates in other cohort HRH studies have ranged from 7.7% in The Nurses and Midwives e-Cohort Study in Australia and New Zealand [25], 19.36% in the Medicine in Australia: Balancing Employment and Life (MABEL) cohort study [31], to 70% in the Longitudinal Analysis of Nursing Education LANE) study in Sweden [45].

We found that the majority of cohort members were women (71.8%), ranging from 62.1% in pharmacy to 100.0% in occupational therapy and oral hygiene (Table 1). In both the medical and pharmacy cohorts, men constituted 35.8% and 37.9% of cohort members respectively. In dentistry, 23.6% of cohort members were male. Historically, these three professions have been male-dominated [46, 47]. In nursing, where gender stereotypes have made it a profession dominated by women, almost one fifth of cohort members (19.1%) were men. Notwithstanding the predominance of men in medicine [48] and physiotherapy [49] in some African countries, the feminisation of the health workforce has been well described [1, 50, 51]. The drivers of this trend include more equal access to training, broader social acceptance, and the humanistic appeal of medicine, among others [19]. This feminisation of the health workforce has implications for career choices and job location decisions. A multi-country study of medical students in 63 medical schools in 11 Latin American countries found that women were less likely to choose general surgery as a specialty [51], while a study in Malawi found that female doctors were less likely to work in rural areas [52]. In Rwanda, a study found that gender stereotypes, and surgical working culture, and lack of professional and personal support influenced women’s decisions to select and/or maintain a career in surgery [53]. WHO has pointed out that female health workers face numerous societal barriers of gender-based violence and sexual harassment in the workplace as well as a greater burden of family responsibilities, which influence their positions in the health hierarchy [1].

Our findings have implications for both the Wits FHS and for South Africa’s health system, in which these graduates will be employed. In the case of the FHS, the curriculum and pedagogical approaches should strive to be gender-transformative, which means that teaching material and patient case studies should be unbiased and not reinforce existing gender stereotypes. Changes in the organisation and culture of health services are also required to adjust to a female-dominated workforce [53]. The lack of women in senior positions and in certain specialties reflects the limited progress that has been made [46]. Special efforts are needed to appoint more women to leadership positions of professions or specialties that have been male-dominated. Mentoring programmes to support women are a proven path to scientific and clinical success, and should be prioritised in the FHS, both in resource allocation and implementation [54].

Although South Africa has made tremendous progress in legislating gender transformation and promoting gender-equity in all policies, the health system could take the lead in making further progress. Strategies could include mainstreaming gender in all health budgets, appointing more women to health leadership positions, providing training opportunities for all health professionals that recognise their care responsibilities, removing discriminatory messages that suggest nursing is only for women, encouraging men to study nursing, and ensuring that the gender-pay gap is eliminated [3, 46].

The ongoing use of apartheid imposed racial categories is contested in South Africa [55–57] yet the country’s apartheid legacy and racism continue to shape socio-economic status, and access to social services. We dealt with our ambivalence about race by asking cohort members how they would identify themselves. Almost one in two (46.1%) of cohort members identified themselves as black African. This illustrates the changing demographic profile of health professional graduates at Wits and other universities in South Africa [40, 58]. For example, unpublished medical school admissions data from Wits show that only 8% of admitted students were black African in 1992 compared to more than 40% in recent years. However, the proportion of black African graduates ranged from a low of 6.1% in occupational therapy to 93.0% for clinical associates. Existing evidence suggests that racial or ethnic diversity of the health care workforce facilitates the provision of responsive and culturally competent care [59, 60]. South Africa’s diverse population requires health professionals that are both representative of and responsive to such diversity. Hence, more efforts are needed in the Wits FHS to ensure the selection of students in occupational therapy, dentistry, and physiotherapy matches the demographic characteristics of the South African population. Some studies have shown that role models and previous exposure or knowledge of a profession influence career choice of health professional students [49, 53, 61]. Strategies to increase diversity in these under-represented professions could include targeted outreach to schools, motivational talks by black alumni who could serve as role models, and partnerships with non-governmental organisations with proven success in recruiting and supporting health professional students from rural and historically disadvantaged areas [62].

In concert with South African government initiatives to redress the geographical inequities in the distribution of health professionals, Wits University has introduced several changes in the admission and selection criteria for health professional students [63]. The 2014 admissions policy aims to reflect diversity of race, gender, socio-economic background, geographic origin, culture, disability, religion, sexual orientation, and national origin [63]. For example, it is envisaged that 20% of places in the medical programme would be offered to top performing rural learners, and another 20% to top performing learners from quintile 1 and 2 schools [64].

A minority of WiSDOM cohort members (13.1%) were born in a rural area, ranging from 0.0% for dentists to 29.3% for pharmacists. A minority had also completed their primary (11.9%) and secondary schooling (8.0%) in a rural area. The study findings could be explained by the cohort effect, as all these graduates would have commenced their studies before the 2014 admissions policy was implemented, and hence the changes introduced by the revised policy would not be evident. Rural origin is important, as several studies have shown that rural backgrounds of health professional graduates are a predictor of their decisions to practice in rural locations [65–67]. Therefore, the small percentage of students with rural backgrounds in all fields of study (Table 1) may mean that they would be less likely to choose to work in rural or under-served settings, where the health needs are greatest in South Africa [68, 69].

In the WiSDOM study, we computed a socio-economic status index of cohort members (Table 2). The highest proportion of relatively privileged individuals from Quintiles 3–5 were from the occupational therapy, physiotherapy, medical and dental groups. This is in contrast to the almost three quarters of oral hygienists, and clinical associates, and the more than half of nurses and pharmacists who came from Quintiles 1–2. Graduates from these four professions were also more dependent on NSFAS funding, compared to their counterparts from Quintiles 3–5. Although the SES index only applies to our cohort study, the relative inequalities in SES among cohort member are a reflection of the inequalities in South African society [70]. Our findings also explain the active participation of many health science students in the 2015 and 2016 #FeesMustFall protests that advocated for affordable higher education in South Africa [71], as they identified with the complex socio-economic, cultural, ideological and political problem enunciated in the protests. While the solutions to these inequalities are complex, our findings suggest that the Wits FHS should strengthen student financial support systems, and examine mechanisms for cross-subsidisation from relatively privileged students to those in need of financial and other support.

WiSDOM found that 29.4% of dental graduates had financed their studies through some kind of loan. A 2017 US study found that education debt influenced career choice, and was associated with the dentists’ decisions to specialise [72]. A study that examined whether educational debt was associated with medical graduating residents’ practice (ownership and type) and fellowship intentions found that those with high debt were less likely to choose government employment or academic practice [73]. However, career choices are complex, and involve numerous factors including intellectual challenge, desire to serve and employment opportunities [74].

In our study, a combination of reasons influenced WiSDOM members to study in the health sciences, with “helping others” and “making a difference in communities” scoring high. The financial reasons “it pays good money” scored high among the high paying professions of dentistry (94.1%) and medicine (75.9%), but very low for nursing (4.8%), which is not surprising given the differences in salaries. For example, the starting salary in the public sector for a junior medical intern is 2.4 times higher than that of a professional nurse, and just slightly higher than that of the most senior professional nurse [75]. Theories that explain choice of career are numerous, and range from those focusing on individual personalities to social systems theories [76]. In a seminal 1968 review that compared different career choice and development theories, Osipow concluded that there was no superior theory, but that the choice and application of a theory were context-specific [76]. Similarly, there is a plethora of studies that explore the reasons why individuals choose different health professions. The reasons are manifold and differ across professions, but include humanitarian (serving the poor and under privileged); societal (prestige, job security, financial security) and scientific (school performance or grades, interest in science, work independence) [77–80].

The relatively low retention rates expressed among nurses, clinical associates and oral hygienists are of concern, and may be related to the finding that these were not their first choice of careers. Nurses are critical to the achievement of UHC reforms in South Africa. Nursing education reforms being implemented in South Africa will require a baccalaureate degree to register as a professional nurse and moving their training from colleges to universities [81]. It is not cost-effective to train highly skilled nurses when half of them state that they plan to leave the profession completely. This implies that the FHS selection criteria should be complemented with interviews and other more innovative selection methods to ensure that the majority of nursing students admitted plan to stay in the nursing profession [82, 83]. The cadre of clinical associates was introduced as one of the measures to increase the number of health workers in under-served areas. In our study, fewer than half (43.2%) plan to stay in the profession. This might be linked to the broader issue of the lack of career paths for clinical associates. The motivations for introducing mid-level workers in South Africa remain valid but government support for this initiative has wavered. Clearer policy direction and financing are required to realise the potential of the clinical associate category [84].

WiSDOM is the first cohort study to be established focusing on eight professional categories. The extensive consultation followed in the WiSDOM study has overcome the risk of participant refusal, with baseline response rate of around 90%. Follow-up of the WiSDOM cohort will generate new knowledge about these dynamics, with important policy implications for the selection and training of health professional students. The baseline descriptive results already raise relevant questions. For example, the FHS should encourage a debate on whether students should be admitted to a degree programme when it is not their first choice, as that may influence their plans to remain in their professions. The new entrants to the FHS are likely to include a larger proportion of individuals who require financial support for their studies because of their economically disadvantaged backgrounds. Hence, there is also a need for a discourse on institutional culture and support systems, within the broader context of South Africa’s health workforce needs. There is currently little data on the impact of these socio-demographic changes and policies on the career and job location choices of health professionals. The WiSDOM study has been established to begin to evaluate these aspects.

## Conclusion

Hitherto, HRH cohort studies are rare in LMICs, especially in Africa. WiSDOM is the first cohort study of health professionals at a South African or African University. The follow-up data will provide information on a number of important health labour market dynamics. Action on the findings of this study will be of particular importance in light of the global emphasis on universal health coverage, and the importance of the health workforce in reaching this ambitious goal.

## References

[1] WHO. Global strategy on human resources for health: Workforce 2030. Geneva: World Health Organization, 2016.

[2] WHO. Draft thirteenth general programme of work, 2019–2023. Report by the Director-General. World Health Assembly Seventy-first World Health Assembly, 71/4, Provisional agenda item 11.1. Geneva: World Health Organization, 2018.

[3] High-Level Commission on Health Employment and Economic Growth. Working for health and growth: investing in the health workforce. Report of the High-Level Commission on Health Employment and Economic Growth. Geneva: World Health Organization, 2016.

[4] McPake B, Maeda A, Araújo EC, Lemiere C, El Maghraby A, Cometto G. Why do health labour market forces matter? Bull World Health. 2013;91:841–6. 10.2471/BLT.13.118794 PMC3853955 24347708

[5] Global Health Workforce Alliance, WHO. A universal truth: No health without a workforce. Geneva: World Health Organization; 2013.

[6] Van Rensburg HCJ. South Africa’s protracted struggle for equal distribution and equitable access–still not there. Human Resources for Health. 2014;12: 26 http://www.human-resources-health.com/content/12/1/26. 10.1186/1478-4491-12-26 24885691 PMC4029937

[7] Rispel LC, Padarath A, editors. South African Health Review 2018. Durban: Health Systems Trust; 2018.

[8] Munyewende P, Rispel LC. Using diaries to explore the work experiences of primary health care nursing managers in two South African provinces Glob Health Action. 2015;7:25323 - 10.3402/gha.v7. PMC4275646 25537937

[9] Armstrong SJ, Rispel LC, Penn-Kekana L. The activities of hospital unit managers and quality of patient care in South African hospitals: A paradox? Glob Health Action. 2015;8:26243– 10.3402/gha.v8.26243 25971397 PMC4430688

[10] The Presidency SA. Twenty year review South Africa, 1994–2014. Pretoria: Government Printing Works; 2014.

[11] NDoH. National Health Insurance Policy: Towards universal health coverage. Pretoria, Republic of South Africa: National Department of Health (NDoH), 2017.

[12] NDoH. National Health Insurance Bill 2019 Government Gazette. 2019;42598:1–60.

[13] Rispel LC, Barron P. Valuing human resources: Key to the success of a national health insurance system. Development Southern Africa. 2012;29(5):616–35. 10.1080/0376835X.2012.730974

[14] Daviaud E, Chopra M. How much is not enough? Human resources requirements for primary health care: a case study from South Africa. Bull World Health. 2008;86(1):46–51. 10.2471/BLT.07.042283 PMC2647342 18235889

[15] Ditlopo P, Blaauw D, Rispel L, Thomas S, Bidwell P. Policy implementation and financial incentives for nurses in two South African provinces: A case study on the occupation specific dispensation Global Health Action. 2013;6(19289 - 10.3402/gha.v6i0.1). PMC3556712 23364085

[16] Heinen MM, van Achterberg T, Schwendimann R, Zander B, Matthews A, Kozka M, et al Nurses' intention to leave their profession: a cross sectional observational study in 10 European countries. Int J Nurs Stud. 2013;50(2):174–84. 10.1016/j.ijnurstu.2012.09.019 . 23107005

[17] Rispel LC, Blaauw D, Chirwa T, de Wet K. Factors influencing agency nursing and moonlighting among nurses in South Africa. Glob Health Action. 2014;7(23585):10.3402/gha.v7.23585. PMC3959456 24647129

[18] Silvestri DM, Blevins M, Afzal AR, Andrews B, Derbew M, Kaur S, et al Medical and nursing students’ intentions to work abroad or in rural areas: a cross-sectional survey in Asia and Africa. Bull World Health Organ. 2014;92 10.2471/blt.14.136051 25378729 PMC4208487

[19] Hossain P, Gupta RD, YarZar P, Jalloh MS, Tasnim N, Afrin A, et al ‘Feminization’of physician workforce in Bangladesh, underlying factors and implications for health system: Insights from a mixed-methods study. PLOS ONE. 2019;14(1):e0210820 10.1371/journal.pone.0210820 30633775 PMC6329528

[20] Goel S, Angeli F, Dhirar N, Sangwan G, Thakur K, Ruwaard D. Factors affecting medical students’ interests in working in rural areas in North India—A qualitative inquiry. PLOS ONE 2019;14(1):e0210251 10.1371/journal.pone.0210251 30629641 PMC6328092

[21] Caruana EJ, Roman M, Hernández-Sánchez J, Solli P. Longitudinal studies. Journal of Thoracic Disease. 2015;7(11):E537–E40. 10.3978/j.issn.2072-1439.2015.10.63 26716051 PMC4669300

[22] Driessen J, Settle D, Potenziani D, Tulenko K, Kabocho T, Wadembere I. Understanding and valuing the broader health system benefits of Uganda’s national Human Resources for Health Information System investment. Human Resources for Health. 2015;13:13:49: 10.1186/s12960-015-0036-0 26321475 PMC4553943

[23] Ishijima H, Mapunda M, Mndeme M, Sukums F, Mlay VS. Challenges and opportunities for effective adoption of HRH information systems in developing countries: national rollout of HRHIS and TIIS in Tanzania. Human Resources for Health. 2015;13:48: 10.1186/s12960-015-0043-1 26077600 PMC4477301

[24] Riley PL, Zuber A, Vindigni SM, Gupta N, Verani AR, Sunderland NL, et al Information systems on human resources for health: a global review. Human Resources for Health. 2012;10:7: http://www.human-resources-health.com/content/10/1/7. 10.1186/1478-4491-10-7 22546089 PMC3433380

[25] Turner C, Bain C, Schluter PJ, Yorkston E, Bogossian F, McClure R, et al Cohort profile: The nurses and midwives e-cohort study—A novel electronic longitudinal study. International Journal of Epidemiology 2009;38:53–60. 10.1093/ije/dym294 18202083

[26] Van Weel C. Longitudinal Research and Data Collection in Primary Care. Annals of Family Medicine. 2005;3(Suppl 1):s46–s51. 10.1370/afm.300 15928218 PMC1466952

[27] Ployhart RE, Vandenberg RJ. Longitudinal research: The theory, design, and analysis of change. Journal of Management. 2009;36(1):94–120.

[28] Liu JX, Goryakin Y, Maeda A, Bruckner T, Scheffler R. Global Health Workforce Labor Market Projections for 2030. Hum Resour Health. 2017;15: 11: 10.1186/s12960-017-0187-2 28159017 PMC5291995

[29] Belanger CF, Hennekens CH, Rosner B, Speizer FE. The nurses' health study. American Journal of Nursing. 1978;78(6):1039–40. 248266

[30] Humphreys JS, McGrail MR, Joyce CM, Scott A, Kalb G. Who should receive recruitment and retention incentives? Improved targeting of rural doctors using medical workforce data. Aust J Rural Health. 2012;20(3–10). 10.1111/j.1440-1584.2011.01252.x 22250870

[31] Joyce CM, Scott A, Jeon S-H, Humphreys J, Kalb G, Witt J, et al The ‘Medicine in Australia: Balancing Employment and Life (MABEL)’ longitudinal survey–Protocol and baseline data for a prospective cohort study of Australian doctors’ workforce participation. BMC Health Serv Res. 2010;10: 50:http://www.biomedcentral.com/1472-6963/10/50. 10.1186/1472-6963-10-50 20181288 PMC2837653

[32] Jinhu Li J, Scott A, McGrail M, Humphreys J, Witt J. Retaining rural doctors: Doctors' preferences for rural medical workforce incentives. Social Science & Medicine. 2014;121:56–64. 25306410 10.1016/j.socscimed.2014.09.053

[33] Li J, Galatsch M, Siegrist J, Mu¨ ller BH, Hasselhorn HM. Reward frustration at work and intention to leave the nursing profession—Prospective results from the European longitudinal NEXT study. International Journal of Nursing Studies. 2011;48:628–35. 10.1016/j.ijnurstu.2010.09.011 20947084

[34] Li J, Fu H, Hu Y, Shang L, Wu Y, Kristensen TS, et al Psychosocial work environment and intention to leave the nursing profession: Results from the longitudinal Chinese NEXT study. Scandinavian Journal of Public Health. 2010;38(3):69–80. 21172773 10.1177/1403494809354361

[35] Sawaengdee K, Tangcharoensathien V, Theerawit T, Thungjaroenkul P, Thinkhamrop W, Prathumkam P, et al Thai nurse cohort study: cohort profiles and key findings. BMC Nursing. 2016;15:10: 10.1186/s12912-016-0131-0 26893589 PMC4757969

[36] Ditlopo P, Blaauw D, Lagarde M. A Longitudinal Study of the Job Choices of a Cohort of South African Nurses to inform Human Resource Policy Interventions: Working Paper #10. Johannesburg: Centre for Health Policy, School of Public Health, University of the Witwatersrand, 2016.

[37] University of the Witwatersrand. Vision 2022: Strategic Framework: Wits-knowledge at the leading edge-concept paper. Johannesburg: University of the Witwatersrand, 2010.

[38] University of the Witwatersrand. 2017 Annual Report of the University of the Witwatersrand incorporating reports of Senate and Council. Johannesburg: University of the Witwatersrand, 2018.

[39] Munro K. Wits Medical School: History, heritage, pioneers and buildings. Wits Review. 2010:33–40.

[40] Van der Merwe L, Van Zyl G, Gibson ASC, Viljoen A, Iputo J, Mammen M, et al South African medical schools: current state of selection criteria and medical students’ demographic profile. South African Medical Journal. 2016;106(1):76–81. 10.7196/SAMJ.2016.v106i1.9913 26792312

[41] WiSDOM. Wits health professional cohort study 2019. Available from: https://www.wits.ac.za/wisdom/.

[42] Wits Faculty of Health Sciences. https://www.wits.ac.za/health/academic-programmes/undergraduate-programmes/ 2019 [cited 2019 9 June ].

[43] Harris PA, Taylor R, Thielke R, Payne P, Gonzalez N, Conde JG. Research electronic data capture (REDCap)—A metadata-driven methodology and workflow process for providing translational research informatics support,. J Biomed Inform. 2009;42(2):377–81. 10.1016/j.jbi.2008.08.010 18929686 PMC2700030

[44] Department of Higher Education and Training. Guidelines for the Department of Higher Education and Training Bursary Scheme for Students at Public Universities. Pretoria: Department of Higher Education and Training, 2019.

[45] Rudman A, Omne-Pontén M, Wallin L, Gustavsson PJ. Monitoring the newly qualified nurses in Sweden: the Longitudinal Analysis of Nursing Education (LANE) study. Human Resources for Health 2010;8(10):http://www.human-resources-health.com/content/8/1/10. 10.1186/1478-4491-8-10 PMC2880980 20423491

[46] WHO. Delivered by women, led by men: A gender and equity analysis of the global health and social workforce. Geneva: World Health Organization, Human Resources for Health Observer Series No. 24, 2019.

[47] Adams T. Gender and feminization in health care professions. Sociology Compass. 2010;4(7):454–65.

[48] Kansayisa G, Yi S, Lin Y, Costas-Chavarri A. Gender-based analysis of factors affecting junior medical students' career selection: addressing the shortage of surgical workforce in Rwanda. Human resources for health. 2018;16(1):29–. 10.1186/s12960-018-0295-7 . 29996860 PMC6042316

[49] Mkondo T, Mudzi W, Mbambo NP. Factors influencing Zimbabwean physiotherapy students in choosing physiotherapy as a career. S Afr J Physiotherapy. 2007;63(3):26–31.

[50] Khan T, Thomas LS, Naidoo S. Analysing post-apartheid gender and racial transformation in medical education in a South African province. Global Health Action. 2013;6(1):19810. 23364091 10.3402/gha.v6i0.19810 PMC3556715

[51] Ng-Sueng LF, Vargas-Matos I, Mayta-Tristán P, Pereyra-Elías R, Montenegro-Idrogo JJ, Inga-Berrospi F, et al Gender Associated with the Intention to Choose a Medical Specialty in Medical Students: A Cross-Sectional Study in 11 Countries in Latin America. PloS one. 2016;11(8):e0161000-e. 10.1371/journal.pone.0161000 . PMC4982605 27519055

[52] Mandeville KL, Ulaya G, Lagarde M, Gwesele L, Dzowela T, Hanson K, et al Early career retention of Malawian medical graduates: a retrospective cohort study. Tropical Medicine & International Health. 2015;20(1):106–14. 10.1111/tmi.12408 PMC4737132. 25329519 PMC4737132

[53] Yi S, Lin Y, Kansayisa G, Costas-Chavarri A. A qualitative study on perceptions of surgical careers in Rwanda: A gender-based approach. PLOS ONE 2018;13(5):e0197290 10.1371/journal.pone.0197290 29746556 PMC5944995

[54] Lescano AG, Cohen CR, Raj T, Rispel L, Garcia PJ, Zunt JR, et al Strengthening mentoring in low-and middle-income countries to advance global health research: an overview. American Journal of Tropical Medicine and Hygiene. 2019;100(1_Suppl):3–8. 10.4269/ajtmh.18-0556 30430982 PMC6329352

[55] Erasmus Z. Confronting the categories: Equitable admissions without apartheid race classification. South African Journal of Higher Education. 2010;24(2):244–57.

[56] Erasmus Z. Apartheid race categories: daring to question their continued use. Transformation: Critical Perspectives on Southern Africa. 2012;79(1):1–11.

[57] Erasmus Z, De Wet J. Not naming race: some medical students' perceptions and experiences of 'race' and racism at the Health Sciences Faculty of the University of Cape Town. Cape Town: University of Cape Town; 2011.

[58] Lehmann U, Andrews G, Sanders D. Change and innovation at South African medical schools. An investigation of student demographics, student support and curriculum innovation. South African Health Review. 2000:10–1.

[59] Cohen JJ, Gabriel BA, Terrell C. The Case for diversity in the health care workforce. Health Affairs. 2002;21(5):90–102. 10.1377/hlthaff.21.5.90 . 12224912

[60] Van Rensburg HCJ. Health and health care in South Africa. Pretoria: Van Schaik; 2012.

[61] Wu LT, Wang W, Holroyd E, Lopez V, Liaw SY. Factors deterring dentistry, medical, pharmacy, and social science undergraduates from pursuing nursing as a healthcare career: a cross-sectional study in an Asian university. BMC Medical Education. 2018;18(1):23 10.1186/s12909-018-1118-1 29373973 PMC5787325

[62] MacGregor RG, Zihindula G, Ross AJ. A rural scholarship model addressing the shortage of healthcare workers in rural areas In: Rispel L, Padarath A, editors. South African Health Review 2018. 2018. Durban: Health Systems Trust; 2018 p. 51–7.

[63] University of the Witwatersrand. Admissions Policy. Johannesburg: University of the Witwatersrand, 2017.

[64] University of the Witwatersrand. WITS University revises its MBBCH degree admissions policy. Johannesburg: University of the Witwatersrand, Faculty of Health Sciences, nd.

[65] Budhathoki SS, Zwanikken PAC, Pokharel PK, Scherpbier AJ. Factors influencing medical students' motivation to practise in rural areas in low-income and middle-income countries: a systematic review. BMJ open. 2017;7(2):e013501–e. 10.1136/bmjopen-2016-013501 . 28232465 PMC5337703

[66] Laven G, Wilkinson D. Rural doctors and rural backgrounds: how strong is the evidence? A systematic review. Australian Journal of Rural Health. 2003;11(6):277–84. 14678410 10.1111/j.1440-1584.2003.00534.x

[67] MacQueen IT, Maggard-Gibbons M, Capra G, Raaen L, Ulloa JG, Shekelle PG, et al Recruiting Rural Healthcare Providers Today: a Systematic Review of Training Program Success and Determinants of Geographic Choices. Journal of General Internal Medicine. 2018;33(2):191–9. Epub 2017/11/27. 10.1007/s11606-017-4210-z . 29181791 PMC5789104

[68] Rural Health Advocacy Project. Rationale for rural proofing the staffing norms Johannesburg: Rural Health Advocacy Project (RHAP), 2013.

[69] Rural Health Advocacy Project. Annual Community Service Officer Allocation Update ‘ACUTE’ Johannesburg: Rural Health Advocacy Project (RHAP), 2018.

[70] World Bank. Overcoming poverty and inequality in South Africa: An assessment of drivers, opportunities and constraints. Washington D.C.: The World Bank, Retrieved from http://documents.worldbank.org/curated/en/530481521735906534/pdf/124521-REV-OUO-South-Africa-Poverty-and-Inequality-Assessment-Report-2018-FINAL-WEB.pdf, 2018.

[71] CMoloi K, Makgoba MW, Ogutu Miruka C. (De)constructing the #FeesMustFall Campaign in South African Higher Education. Contemporary Education Dialogue. 2017;14(2):211–23. 10.1177/0973184917716999

[72] Nasseh K, Vujicic M. The relationship between education debt and career choices in professional programs: The case of dentistry. The Journal of the American Dental Association. 2017;148(11):825–33. 10.1016/j.adaj.2017.06.042 28843498

[73] Phillips JP, Peterson LE, Fang B, Kovar-Gough I, Phillips RL. Debt and the Emerging Physician Workforce: The Relationship Between Educational Debt and Family Medicine Residents’ Practice and Fellowship Intentions. Academic Medicine. 2019;94(2):267–73. 10.1097/ACM.0000000000002468 30256252

[74] Puertas EB, Arósquipa C, Gutiérrez D. Factors that influence a career choice in primary care among medical students from high-, middle-, and low-income countries: a systematic review. Revista Panamericana de Salud Pública. 2013;34:351–8. 24553763

[75] Department of Public Service Administration. Salary scales, with translation keys, for employees on salary levels 1 to 12 and those employees covered by Occupation Specific Dispensations (OSDs). Pretoria: DPSA, 2018.

[76] Osipow SH. Theories of Career Development. A Comparison of the Theories. New York: Appleton-Century-Crofts; 1968.

[77] Goel S, Angeli F, Dhirar N, Singla N, Ruwaard D. What motivates medical students to select medical studies: a systematic literature review. BMC Medical Education. 2018;18(1):16 10.1186/s12909-018-1123-4 29343262 PMC5772649

[78] Marcinowicz L, Owlasiuk A, Slusarska B, Zarzycka D, Pawlikowska T. Choice and perception of the nursing profession from the perspective of Polish nursing students: a focus group study. BMC Medical Education. 2016;16(1):243–. 10.1186/s12909-016-0765-3 . 27644123 PMC5029103

[79] Hanna L-A, Askin F, Hall M. First-Year Pharmacy Students’ Views on Their Chosen Professional Career. American Journal of Pharmaceutical Education. 2016;80(9):150 10.5688/ajpe809150 28090099 PMC5221832

[80] Avramova N, Yaneva K, Bonev B. First-year dental students’ motivation and attitudes for choosing the dental profession. Acta medica academica. 2014;43(2):113–21. 10.5644/ama2006-124.110 25529516

[81] Blaauw D, Ditlopo P, Rispel LC. Nursing education reform in South Africa: lessons from a policy analysis study. Global Health Action. 2014;7:26401 - 10.3402/gha.v7. PMC4275647 25537941

[82] Traynor M, Galanouli D, Roberts M, Leonard L, Gale T. Identifying applicants suitable to a career in nursing: a value-based approach to undergraduate selection. Journal of Advanced Nursing. 2017;73(6):1443–54. 10.1111/jan.13227 27905663

[83] Patterson F, Prescott-Clements L, Zibarras L, Edwards H, Kerrin M, Cousans F. Recruiting for values in healthcare: a preliminary review of the evidence. Advances in Health Sciences Education. 2016;21(4):859–81. 10.1007/s10459-014-9579-4 25616718

[84] Couper I, Hugo J. Addressing the shortage of health professionals in South Africa through the development of a new cadre of health worker: the creation of Clinical Associates. Rural & Remote Health. 2014;14(3). 25130766

